# 207 Early Impact of a Jurisdiction?Led Antimicrobial Stewardship Feedback Intervention Using NHSN SAAR Data Across Acute Care Hospitals

**DOI:** 10.1017/ash.2026.10593

**Published:** 2026-06-23

**Authors:** Holly Maples, Lorelei Kellhofer, Taylor Bandala, Rachel Frenner, Geoff Curran

**Affiliations:** 1 UAMS College of Pharmacy & Arkansas Children’s; 2 Arkansas Children’s Hospital/University of Arkansas for Medical Sciences; 3 Arkansas Children’s Hospital

## Abstract

**Background** Implementation of diagnostic stewardship is a key strategy in antimicrobial stewardship to improve use of antimicrobials and human resources to promote value-based care. Pediatric urinary tract infections represent the most common pediatric infection with increasing antibiotic resistance and high utilization of diagnostic testing. The purpose of this study is to examine the impact of implementing a reflex urine culture (UCx) algorithm to reduce unnecessary UCxs in a pediatric emergency department. **Methods:** This is a pre/post study from April 1st, 2022 through March 31st, 2025, with the UCx algorithm intervention implemented in July 2023. A urinalysis (UA) and UCx report was generated from EPIC, placed in Excel, and analyzed descriptively and statistically via Wizard 2.0, Version 2.0.14. Data included age, gender, weight, race, ethnicity, payor status, language, zip code, and UA and UCx results. Children 2-18 years of age were included. Patients <2 years of age and any patient with only a point of care test (POCT) UA, UCx, or urine microscopic test, or duplicate test within 24 hrs were excluded. The reflex criteria was defined as a UA with ≥5 WBC/hpf and excluded children <2 years of age or who are immunocompromised. Results A total of 17,502 unique patients with a UA were evaluated. Both pre (n=6,956) and post (n=10,597) were similar for demographics including gender (female 69.7% vs 69.4%) and average age (9.93 years vs 9.97 years). Percent of UA’s with ≥5 WBC/hpf in the pre (n=1,567) vs post (2,361) were also similar at 22.5% vs 22.3% (p=0.7). 3,453 total UCxs were completed in the pre compared to 3,170 post. The UCx to UA ratio was 49.6% pre compared to 29.9% post (p<0.001). An improvement in UCx positivity rate from 11% pre to 20.3% post (p<0.001) was also shown. Cost savings further justifies the intervention as a projected $300,960 was saved by preventin 2,090 unnecessary UCx ($144 /culture). No differences were found by demographics except for race. While all races (black, white, other) saw a significant improvement in urine culture obtainment (p<0.001), white patients had a larger decrease from 48% to 27.2% compared to black from 49.9% to 33.4%. Conclusions A reflex UCx algorithm can be a successful implementation tool in diagnostic stewardship. Next steps will include exploring more stringent reflex criteria, lowering the age to 1 year, assessing the impact on health equity, and evaluating the impact in other health care locations to promote further value-based care.

